# Overcoming biases of individual level shopping history data in health research

**DOI:** 10.1038/s41746-024-01231-4

**Published:** 2024-09-30

**Authors:** Anya Skatova

**Affiliations:** Digital Footprints Lab & Medical Research Council Integrative Epidemiology Unit at the University of Bristol, Population Health Science, Bristol Medical School, University of Bristol, Bristol, UK

**Keywords:** Epidemiology, Public health

## Abstract

Novel sources of population data, especially administrative and medical records, as well as the digital footprints generated through interactions with online services, present a considerable opportunity for advancing health research and policymaking. An illustrative example is shopping history records that can illuminate aspects of population health by scrutinizing extensive sets of everyday choices made in the real world. However, like any dataset, these sources possess specific limitations, including sampling biases, validity issues, and measurement errors. To enhance the applicability and potential of shopping data in health research, we advocate for the integration of individual-level shopping data with external datasets containing rich repositories of longitudinal population cohort studies. This strategic approach holds the promise of devising innovative methodologies to address inherent data limitations and biases. By meticulously documenting biases, establishing validated associations, and discerning patterns within these amalgamated records, researchers can extrapolate their findings to encompass population-wide datasets derived from national supermarket chain. The validation and linkage of population health data with real-world choices pertaining to food, beverages, and over-the-counter medications, such as pain relief, present a significant opportunity to comprehend the impact of these choices and behavioural patterns associated with them on public health.

Every day, we leave behind ‘digital footprints’; a trail of data created through our on-line and in-person activities, collected through technology. These ‘digital footprints’ are a reflection of our choices, activities, preferences and habits. The advent of digital technology has transformed comprehension of health and related behaviours, driven by the meticulous collection of these digital footprints across various data repositories^1–5^. This has led to a wealth of research that utilises digital footprints data, for example wearables to track population health indicators^6–8^ and assess the success of interventions^9^; consumer app data analysed to derive prevalence and course of health symptoms in different domains^10,11^ and consumer behavioural data, such as shopping and banking records, being examined to derive predictors of life outcomes including health and well-being^12–15^.

Shopping data are collected when we engage in supermarket transactions and use loyalty cards (known as club cards in USA and other countries) to get future benefits, such as discounts. In this process, supermarkets record our purchases to better understand their consumers to maximise profits. While collected for commercial purposes, shopping data can be uniquely beneficial to public health research because it can provide a comprehensive portrait of our consumption habits and preferences.

Conventional approaches to scrutinizing the interplay between lifestyle choices, health behaviours and outcomes rely heavily on self-report questionnaires, wherein individuals are asked to recollect and/or recount their daily choices and behaviours (e.g., in the areas of diet, tobacco and alcohol consumption^16,17^). Such a methodological approach contains potential biases, as responses are frequently coloured by memory lapses or socially desirable responses that may not faithfully mirror actual behaviours^18,19^. However, shopping history data—while not without biases itself—provides a different insight into people’s behaviours by cataloguing real world choices related to consumption and behaviours, in real time. These datasets hold the potential to provide a new, additional window into people’s lives helping to elucidate the intricate connections between everyday behaviours and lifestyle choices on one hand, and their profound impact on health and social outcomes on the other.

Historically, access to these datasets was confined to private enterprises, primarily harnessing the data for tracking sales trends, deciphering market dynamics, and optimizing targeted marketing strategies. However, a shift in data protection legislation in the EU, namely the General Data Protection Regulation (GDPR), opened new possibilities by granting the public the ability to access and contribute their data for academic research^20,21^. Individual level shopping data can now be shared with research organisations by individuals requesting access to the data collected about them. This unprecedented access path is transformative given the promise this data holds for population health research^22–27^.

In this paper we explore how individual-level shopping data can contribute to health research, discuss their potential for understanding the interplay between health and consumption patterns, and suggest ways these could be best utilised by overcoming potential biases in the data.

## The value of shopping data for health research

Shopping data can serve as a distinctive vantage point for exploring intricate queries in population health research. It enables researchers to unveil nuanced behavioural patterns and correlations that could be overlooked due to biases in traditional survey-based methodologies^17,19,28^; for example, participants under reporting or misremembering their habits or choice patterns. This is particularly important given behavioural risk factors like diet, alcohol, and tobacco consumption significantly predict a wide range of health outcomes, including diabetes, cancer, heart disease, and mental health issues^29–31^. For instance, smoking is strongly associated with cancer^32^, certain dietary patterns influence cancer outcomes^33^, and increased alcohol consumption leads to cardiovascular issues and reduced life expectancy^34^.

Shopping records a spectrum of behaviours, ranging from dietary choices and alcohol purchases to gambling tendencies and pain and weight management patterns. They can contain information that provides the possibility for new insights into how diet, alcohol and tobacco consumption, medication of symptoms of various illnesses of the whole populations are related to health outcomes (see Box 1 for a limited number of exemplary study vignettes). Moreover, purchasing patterns, such as over-the-counter medication, can serve as early indicators of underlying health conditions^22,35^.

Loyalty cards provide a detailed record of individuals’ purchasing history and offer an avenue for tracking expenditure over extended periods, often spanning decades. This archival depth facilitates the acquisition of multiple data points per individual cardholder, providing a fine grain timeline of transactions. These data provide researchers with a source of granular, objective, detailed insights into people’s everyday choices and behaviours across diverse domains^5,23,36^, including population diet^37^ and outbreaks investigation^38^. In turn, it can contribute to the development of more targeted public health interventions that could enhance the health and well-being of communities.

Box 1 What can we find out from loyalty cards for health research?

**Table:** 

Alcohol consumption and health	Ready meals and obesity	Household products and health risks in children
Shopping data can be instrumental in studying the levels of alcohol consumption that pose risks to health. Alcohol consumption is known to be severely underpotted in the surveys. By analysing loyalty card records, researchers can identify purchasing patterns of alcoholic beverages of different individual card holders. This data, combined with individual characteristics like age and socio-economic background, allow for a nuanced analysis of the relationship between alcohol intake and individual decisions. Such insights can inform public health guidelines and interventions to minimize the risk of negative health outcomes.	Understanding the role of ready meals in contributing to obesity is a multifaceted challenge. Shopping data provides a unique opportunity to investigate this issue. Researchers can track the frequency and types of ready meals purchased by individuals, considering factors such as age, gender, and household composition. In addition, the data can be correlated with nutritional information to assess the impact of specific meal choices on weight gain. This research can uncover the conditions under which ready meals are most likely to lead to obesity, guiding dietary recommendations and obesity prevention strategies.	Shopping data can be a valuable resource for exploring the potential health risks posed by chemicals in household products, particularly in children. Researchers can examine loyalty card records to identify households that consistently purchase products containing certain chemicals. This information is only available because of existing product descriptions and very hard to achieve through surveys. It can contribute to better understanding health outcomes in children, such as respiratory conditions or skin irritations, uncovering the association between exposure to specific chemicals and adverse health effects. This research can inform safety guidelines and product labelling to protect children from potential harm.

## Limitations of shopping data

While shopping data offers valuable insights, it is not without limitations. These limitations include data privacy concerns (which are outside the scope of this paper although see refs. ^24,39,40^ for discussion of some of the key concerns), as well as potential biases in data collection and data quality issues. For example, loyalty card data may not capture the entire spectrum of consumer behaviour and may exclude purchases made without loyalty card usage (such as takeaway meals). Further, the exact (micro)-nutrient content of many products (e.g., ready meals) might not be readily available from supermarket or other databases creating difficulty for analysis and comparison across different supermarkets. Demographic, and socio-economic biases may exist in loyalty card holders, limiting the representativeness of the data. To understand how to effectively utilise the resource of shopping data for health research, we need to identify biases in shopping data, as well as how the patterns in the shopping data are linked to health outcomes.

There are several main methodological challenges in using shopping data to study health. First challenge related to *who* we are measuring (i.e., sampling biases). Second is *what* we are measuring (i.e., validity); and third is what we are *not* measuring and *why* (i.e., measurement error). To achieve the benefits that shopping data can provide while ameliorating biases, these data need to be linked to other datasets on individual level.

## Data linkage for enhanced value

By integrating shopping data with other datasets, we can address the methodological challenges associated with sampling biases, measurement errors, and data validity. This approach can be applied in both standalone cross-sectional studies^14,22,37^ as well as within longitudinal population studies’ databanks^24^. Longitudinal population studies are cohorts of individuals followed over years, often decades, with rich biomedical and social data collected at multiple time points. Examples of such studies in UK including Avon Longitudinal Study of Parents and Children (ALSPAC^41^) or The 1970 British Cohort Study^42^.

Shopping data is not immune to sampling biases (see Challenges and Solutions summarised in Box 2), with self-selection being a notable example. It inherently reflects the behaviours of individuals who frequent specific outlets and engage in loyalty card programs. While such data may encompass extensive geographic coverage, particularly with nation-wide supermarkets like Tesco or Sainsbury’s in the UK, it falls short of being a perfectly representative cross-section of the general population. The inherent selection bias arises from both the demographic characteristics of individuals who shop at specific outlets and those who willingly enrol in loyalty card schemes. Consequently, a critical evaluation of loyalty card data is essential to gauge its fidelity in representing the intended population^43^.

To mitigate these biases, one approach involves linking shopping data with cross-sectional or longitudinal samples known for their demographic characteristics, subsequently applying statistical weights to different groups. This procedure can partially rectify the effects of sampling bias through statistical means. A noteworthy example is offered by Vuorinen et al.^43^, who calculated adjustment weights for measurements derived from loyalty card data, particularly within specific demographic or socio-economic groups, using data from a major supermarket chain in Finland.

Moreover, the integration of shopping data into longitudinal population studies presents an opportunity to extract a comprehensive characterization of cohort participants, allowing for the examination of patterns, moderators, and predictors related to consent and the utilization of loyalty cards. It allows to link data from multiple supermarkets for the same participant. Potential moderator variables warranting exploration include the distinction between loyalty card owners and those who do not own loyalty cards but shop at the same outlet, the possession of various loyalty cards for similar supermarkets/retail stores (e.g., Waitrose vs. Sainsburys in the UK), and individuals’ choices regarding data sharing. A deeper understanding of the sources of sampling bias through these linkages of purchase data from different sources provides the foundation for subsequent corrective actions, such as incorporating bias-inducing variables such as covariates or imposing constraints on the interpretation of research findings.

Secondly, within a supermarket shopping context, individuals make purchases that span a spectrum of purposes. These include acquiring items for their entire family, for personal use, or for special occasions, as well as products for immediate or future consumption—or in some cases, products that are never consumed, like purchasing vitamins that remain untouched. Moreover, individuals may purchase products with intentions that deviate from their intended use, such as using laxatives for weight loss. This multiplicity of purchase behaviours introduces measurement errors that can introduce bias when analysing the relationships between shopping patterns and health outcomes.

To pinpoint the sources of measurement error, particularly those related to incomplete behaviour representation based on transaction-derived variables, it is imperative to place these variables in their broader context. For example, assessing what proportion of shoppers buy formula specifically for their babies, or solely from a particular outlet. By collecting self-reports and contextual information, we can gauge whether patterns of certain purchases in the data indeed reflect *actual* behaviours.

To address these issues, direct access to the subset of individuals from whom the data is collected is vital. This facilitates the collection of self-reports that investigate whether the products purchased were consumed, allowing researchers to assess whether contextual factors introduce bias into the results. This can be achieved through various means, such as self-reports in surveys or interviews.

Finally, the significance of patterns within the data hinges on the question of whether loyalty card data truly represents behaviour in specific domains, such as assessing whether a history of alcohol purchases is a valid measure of individual alcohol consumption. Just as with any new source of behavioural information, transaction data necessitates validation. In a manner akin to the rigorous validation process applied to a new questionnaire, it becomes essential to identify which transaction-derived variables exhibit consistent associations with well-established self-report and health metrics. This may entail a triangulation of multiple information sources, all scrutinizing the same behaviour. For example, we might inquire whether individuals displaying purchasing patterns associated with pregnancy, as indicated by transaction data, indeed correspond with pregnant individuals as reported through self-reports. Linking shopping data with cross-sectional or longitudinal studies offers the opportunity to collect self-reports related to targeted health-related behaviours allowing such triangulation. These self-reports, such as predicting the distribution of various food categories in household diets from the distribution of food purchases, can then be used for the validation of transaction-derived variables within linked datasets^26^.

By rigorously validating patterns in shopping history data that serve as reliable indicators of significant health outcomes and behaviours, we can advance methods for integrating and triangulating diverse datasets, including shopping history, medical records, and self-reports, in the realm of health research. Patterns in shopping data which are validated and documented in terms of the sampling biases and measurement error, can be then transferred to the nation-wide anonymous “un-linked” datasets to better understand nation-wide issues around public health and assess public health interventions.

Box 2 Solutions to overcome biases in shopping data

**Table:** 

	Challenges	Solutions
Sampling bias: Who are we measuring?	• Who shops in the supermarket *X*? • Who opts into the loyalty card scheme? • Who consents to share their data?	• Link shopping data within an longitudinal population study allowing analysis of the socio-economic and other moderators’ consent, the choice of the retailer and opting-in into the loyalty cards scheme^24^; • Determine weights to be added for analysis of health outcomes of different groups^43^.
Validity: What are we measuring?	• Do patterns in shopping data represent behaviour(s) of interest? (e.g., does buying more vegetables means consuming more vegetables)	• Triangulate patterns in the shopping data with “gold standard” measures (e.g., additional surveys to match cards data information to self-reports^41^).
Measurement error: What are we not measuring and why?	• Do purchases in category *Y* in supermarket *X* cover all purchases in category *Z*? • What percentage of purchases is recorded through a loyalty card? • How well does a loyalty card represent consumption of an individual vs household?	• Link additional survey responses to gain context (e.g., how regularly do use your loyalty card while shopping at [name of supermarket/retail outlet]; how much shopping with the use of this loyalty card accounts for your overall shopping; out of all your purchases in this outlet, what percent of shopping is for you/others?).

## Linking shopping data into longitudinal population studies

It has been argued that linking shopping data to additional data sources not only aids in unravelling the inherent biases and limitations within shopping datasets but also affords an opportunity to address methodological and quality concerns associated with such datasets. In this regard, linkages to longitudinal population studies represent a particularly rich avenue for exploration. Population studies serve as a unique portal into the lives of thousands of individuals, meticulously accumulating data over the years, yielding a rich tapestry of information which can provide valuable context and depth.

With a wealth of data banks that include social, biomedical, health, and administrative records^41^, they provide a comprehensive foundation for examining issues related to sampling, representativeness, validity, and data quality in shopping datasets. The synergistic approach of linking these datasets allows for the identification of biases, the isolation of outliers, and the development of innovative algorithms to enhance data quality.

In a first of its kind initiative, the integration of shopping data within a longitudinal cohort is presently unfolding in ALSPAC, as reported in detail by Skatova and Boyd^24^. This process encompasses a journey through four distinct stages. The first phase involved an extensive exploration of participant acceptability^40^. Through collaborative efforts, an opt-in consent approach is co-designed with study participants, laying the foundation for a mutually agreeable engagement.

The second step included the technical pilot linkage, a critical juncture where the data pipeline is tested. Commencing with the acquisition of participant consent, the journey progresses through the retrieval of data from industry partners. This shopping data donated by participant and collected by the study from the industry partner has been then embedded within the ALSPAC databank.

The third stage involved actively reaching out to cohort participants for an opt in consent for collecting their shopping history data. This important stage solidified the participatory nature of the endeavour as well allowing the researchers to collect loyalty card details.

Finally, the culmination of efforts is marked by the integration of shopping data donated by participants with study databank. The shopping dataset becomes an important part of the study’s data assets, opening avenues for exploring sampling biases, measurement errors, and validating data patterns. Importantly, this integration capitalizes on the wealth of previously collected data within the ALSPAC, enriching the potential for nuanced insights and comprehensive analyses.

## Conclusion & future directions

The digital age has ushered in the vast collection of digital footprints across various data repositories. Among these repositories, shopping data is a largely untapped resource, offering a unique perspective on understanding complex behavioural patterns at the national level and over long periods of time as well as their impact on health outcomes. Unlike traditional research heavily reliant on self-report questionnaires, shopping history data provides an alternative—complimentary to more traditional methods—lens through which to examine people’s behaviours, unveiling the complex connections between everyday behaviours, lifestyle choices, and their profound influence on health and social outcomes. We have argued however that shopping data is not without its limitations, encompassing data privacy and ethics concerns as well as potential biases in data collection and quality. To fully unlock the potential of shopping data for health research, a comprehensive understanding of these limitations is crucial. Integration of shopping data with other datasets, particularly within longitudinal population studies, can address methodological challenges related to sampling biases, measurement errors, and data validity. These studies, enriched with biomedical and social data, provide a valuable platform for evaluating shopping datasets in light of multifaceted information collected from the population. This approach not only mitigates the limitations of shopping datasets but also transforms them into a powerful tool for robust and insightful research of the complexities of real-life behaviours and their health implications.

Research utilizing well-described and validated shopping data has the potential to bring about a paradigm shift in population health methods, especially in areas such as diet, alcohol, and tobacco consumption, as well as early disease detection and symptom management. Future research in this field should prioritize the refinement of methodologies through data linkages and the expansion of data sources to triangulate patterns within the data, thereby opening new avenues for enhancing population health outcomes.

The utilization of shopping data in conjunction with longitudinal surveys and other observational data has the potential to revolutionize population health research. By leveraging shopping data with ethical rigor and integrating it with other health-related information, we can unlock its potential via a careful consideration of its limitations and biases. This approach can enhance our comprehension of how everyday behaviours impact health outcomes, facilitate the improved identification of lifestyle-related causes and early disease symptoms, enable researchers to assess the impact of national policies, and ultimately provide valuable recommendations for health interventions.
